# Preventing post-discharge suicides in psychiatric patients: insights from patients, lay healthcare supporters, and mental health professionals—a qualitative analysis

**DOI:** 10.1186/s12889-023-17475-w

**Published:** 2024-01-02

**Authors:** Tiantian Fu, Huiming Liu, Chang Chen, Bin Zhang, Guanjie Chen, Yuanhan Bai, Jinghua Li, Fengsu Hou

**Affiliations:** 1 Department of Medical Statistics, School of Public Health, Sun Yat-Sen University, No. 74 Zhongshan 2Nd Road, Guangzhou, 510080 Guangdong China; 2 Sun Yat-Sen University Global Health Institute, Sun Yat-Sen University, Guangzhou, China; 3 Department of Public Health, Shenzhen Mental Health Center/Shenzhen Kangning Hospital, No.1080, Cuizhu Road, Shenzhen, 518020 Guangdong China; 4 Department of Bipolar Disorders, Shenzhen Mental Health Center, Shenzhen Kangning Hospital (Pingshan Campus), Shenzhen, Guangdong Province China

**Keywords:** Post-discharge suicide, Psychiatric patients, Problems and unmet needs, Community-based participatory research, Thematic analysis

## Abstract

**Background:**

Discharged psychiatric patients are at higher risk of suicide due to various risk factors in their lives compared to the general population. However, specific problems and needs of these patients after discharge remain unclear. This research constitutes a segment of a broader implementation study designed to formulate an interventional strategy targeting post-discharge suicide among Chinese psychiatric patients. The present study seeks to qualitatively investigate the problems and needs from the perspectives of patients, their lay healthcare supporters (LHSs), and mental health professionals (MPs), aiming to enhance the efficacy of the interventional strategy.

**Methods:**

This study is part of a larger implementation study based on Shenzhen Kangning Hospital (SKH) in Shenzhen, Guangdong, China. Under the community-based participatory research framework, we recruited discharged psychiatric patients, their LHSs, and MPs as a collaborative community team, and we conducted individual in-depth interviews for patients and LSHs and focus group interviews with MPs. We utilized a thematic analysis approach to identify sub-themes and themes from interviews through systematically coding and analyzing the data.

**Results:**

A total of 45 participants were recruited for interviews, comprising 17 patients, 8 LHSs, and 20 MPs. We conducted 25 individual in-depth interviews and 3 focus group interviews. Through the interviews, we identified three themes of post-discharge problems: problems related to self, family-related problems, societal and community-related problems. We also identified four themes related to reducing post-discharge suicide: proactive self-management, multifunctional relatives, multifunctional MP group, and a warm society. The tangible support from LHSs and emotional support from MPs are strongly emphasized. Follow-up interventions were identified as the most significant way to addressing these unmet needs. Instrumental support from the community and a caring and non-discriminatory environment for individuals with mental disorders are essential for reducing suicide risk.

**Conclusions:**

Establishing an integrated mental health care service network that connects psychiatric patients, LHSs, and MPs cross community and societal sectors, with patient-centered follow-up care at its core, is a practical approach to better address patients’ needs and reduce post-discharge suicide.

**Trial registration:**

Registration number: NCT04907669.

Date of registration: May 26th,2021.

**Supplementary Information:**

The online version contains supplementary material available at 10.1186/s12889-023-17475-w.

## Background

Suicide is a global significant public health issue. According to World Health Organization (WHO), there are approximately 703,000 individuals died by suicide annually worldwide, and the global age-standardized suicide rate is about 9.0 per 100,000 people in 2019 [1]. Global efforts are required to achieve one of the Sustainable Development Goals (SDGs) set by the WHO, which is to decrease one-third of suicide by 2030 [2, 3]. China has made great efforts, and the suicide rate in China dropped to 7.09 and 4.31 per 100,000 people in rural and urban in 2021, respectively [4].

The morality rate of individuals with mental disorders is five times higher than that of the general population, with suicides contributing to 40% of these fatalities [5, 6]. Notably, patients discharged from psychiatric facilities face significant greater suicide risk, and the pooled rate was 484 per 100,000 people in the first 12 months after discharge in UK, which can be up to 2950, 2060 and 1132 per 100,000 people in the first week, the first month and three months after discharge, respectively, according to research in England, Wales and America [7–10]. However, we know of only one study in China reported a 12-month suicide mortality rate of 1062/100 000 among patients discharged from psychiatric facilities [11] In addition to the high prevalence, there is established information about risk factors associated with post-discharge suicide among psychiatric patients. While little is known about the risk factors in the context of China, there have been research worldwide that provide some insights. Commonly reported risk factors include gender, age, education, physical health, history of suicide attempts, etc., and we used the social ecological model to contextualize commonly studied risk factors (see Table 1) [8, 12–30].

**Table 1:** Factors associated with post-discharge suicide among psychiatric patients

Individual level	Interpersonal and communal level	Societal and cultural level
***Demographic factors***	• Social isolation	***Mental health service factors***
• Gender	• Relationship conflicts, discord or loss	• Substandard mental health care during hospitalization
• Age	• Interpersonal violence and/or trauma	• Insufficient mental health service after discharge
• Marriage	• Conflicts with doctors during hospitalization	• Transition to new physicians, psychotherapist, and counselor
• Education	• Financial difficulties	• Treatment regimen
• Income	• Lack of social support	• Availability to social worker service
• Solitary		• Involuntary admission
***Psychiatric illness-related factors***		• Discharge against medical advice
• Psychiatric diagnosis		• Brief hospitalization stay
• Severity of symptoms		• Quality of family-based care
• History of self-harm		***Socio-cultural factors***
• Previous suicide attempts		• Stigma associated with help-seeking behaviors
• Previous psychiatric hospitalization		• Mental health policies
• Re-admission or relapse within 3 months		• Social welfare
• Illness duration < 12 months		• Access to means
***Others***		• Inappropriate media reporting and social media use
• Physical illness and chronic disabilities		
• Work-related stress		
• Unemployment		
• Adverse life events		
• Difficulties in adapting to a new environment		
• Regimen compliance		
• Tolerance to side effects		

Existing studies on suicide underscores the premise that suicidal behaviors are frequently driven by intense emotional states perceived as intolerable by individuals which can be described by a spectrum of terms including anguish, desperation, mental pain, emotional dysregulation, annihilation anxiety, and isolation [31–35]. Though some studies indicated that hospitalization could provide temporary relief from these feelings, it does not necessarily reduce suicide risk in the long term [36]. Patients often experience existential angst and dread upon discharge, as they are faced with the daunting prospect of leaving the supportive environment of the hospital and returning to their daily lives. They may feel disconnected from others and uncertain about their abilities to cope with stressors that may have contributed to suicide attempts, challenges that may reintegrate into their regular lives, possible relapses in the future, and stigma related to mental health problems [36–38]. Thus, it is important to implement structured transitional mental health care services to support patients after psychiatric admissions [39].

Evidence-based guidelines recommend integrated interventions to reduce post-discharge suicide among psychiatric patients, which involve suicide risk assessment, safety planning, medication, psychological and behavioral therapy, regular follow-up, health education, emergency intervention system, and collaborative care from lay healthcare supporters (LHSs) and mental health professionals (MPs) [40–42]. However, systematically implementing the interventions requires adequate mental health care service resource. Given there were only about 5.47 psychiatric professionals per 10,000 people in China, compared to 38.78 in Korea, 11.9 in Japan and 10–20 in many European countries [43, 44]. For individuals with severe mental disorders including schizophrenia, schizoaffective disorder, paranoid psychosis, bipolar disorder, psychotic disorders due to epilepsy or intellectual developmental disorder with psychotic disorders, they will be rated from levels 0–5 for the risk of violent behaviors and will receive follow-ups from community mental health workers. The frequency of the follow-ups will be weekly, bi-weekly and monthly based on the ratings; and the follow-ups is primarily to prevent violent behaviors towards the public rather than post-discharge suicide [45]. For individuals with other mental disorders, they will receive follow-ups from community mental health service providers only if they are identified as suicide risks by clinical physicians in the management system. Patients not flagged for suicide risk will need to proactively initiate contact with mental health intervention personnel to access suicide risk interventions [4, 45].

Thus, it’s important to develop and implement interventions adapted to the context in China. Further, we notice that most interventions are designed or implemented from a top-down approach, rather than being patient-centered, and only a few have considered the problems that patients may face after discharge and their needs [46, 47]. Hence, to help develop patient-centered policies and interventions to reduce post-discharge suicide risk among psychiatric patients, this study aims to explore problems faced by patients after discharge and their needs from the perspectives of patients and lay healthcare supporters who are usually patients’ family members and friends. Additionally, the study will incorporate insights from mental health care providers and policymakers, whose input is vital for improving intervention quality and efficacy, and for promoting a collaborative environment of empowerment, engagement, and trust between service users and providers.

## Methods

### Study setting

This study is part of an implementation study that aims to apply a text-based brief contact intervention to reduce post-discharge suicide among psychiatric patients from Shenzhen Kangning Hospital (SKH) in Shenzhen, Guangdong, China, and evaluate its implementability [48]. SKH is the only public psychiatric hospital that provides in-patient psychiatric services in Shenzhen City with over 1500 in-patient beds, and the hospital had 15,043 in-patient stays and 571,000 out-patient visits in 2021. In the preliminary study, out of the 520 hospitalized patients with initial suicide risk at admission in SKH who participated in post-discharge follow-up surveys, our subsequent telephone follow-ups between April 1st and June 31st, 2019 revealed 5 cases of post-discharge suicide (3 males and 2 females). The suicide rate among discharged patients within three months was 961.54 per 100,000, markedly surpassing the general suicide rate in the Chinese population by over 140 times. During hospitalization, SKH provides environment balances therapeutic needs with safety, involving multidisciplinary teams for treatment. Families and mental health social workers are welcomed to collaborate with clinic professionals. The mean duration of hospitalization in SKH spans 30 days. Noted, as this study was conducted during the COVID-19 pandemic, family visits were limited that each patient was allowed one visit from family members per day in accordance with COVID-19 policies.

### Sample and sampling

Under the community-based participatory research (CBPR) framework, this study aimed to recruit discharged psychiatric patients from SKH, their lay healthcare supporters (LHSs) who are usually family members, and mental health professionals (MPs) as the community team [49, 50]. There were two types of MPs: 1) clinic psychiatrists, nurses and psycho-crisis intervention team members from SKH, and community mental health workers and mental health social workers from eight community health centers in Shenzhen.

We conducted purposive sampling to recruit participants for the community team. We recruited participants who learned about our study through different channels and volunteered to participate, including patients and LHSs recommended by doctors, and colleagues recommended by MPs who were willing to participate. The recruited personnel are full-time scientific researchers and research assistants for SKH, and non-ward clinical staff. There are no conflicts of interest between the recruiters and the recruited. Sample size was ascertained based on the principle of theoretical saturation. After interviewing, we encoded the obtained audio materials immediately. The interview process concluded when newly acquired audio materials failed to elicit unique codes, indicating the achievement of theoretical saturation [51]. The parent study aimed to conduct both in-depth and focus group interviews among all groups of participants, however, due to the regulations for COVID-19 prevention and control in SKH, we could only conduct in-depth interviews with patients and LHSs, and focus group interviews with MPs [48]. The inclusion criteria for MPs were: (1) being 18 years and above and (2) having practiced in mental health service for at least 12 months. The inclusion criteria for patients are: (1) being 18 years and above, (2) being diagnosed with psychotic symptoms or Major depressive disorder (MDD) based on the ICD-10 [52], (3) having received inpatient care for 3 days or more, (4) living in Shenzhen and having no plan to leave Shenzhen in the following 12 months after discharge, and (5) being able to read text messages, answer phone calls on mobile phones, use WeChat or any application on smart phones. And the inclusion criteria for LHSs are: (1) being 18 years and above, (2) without diagnosis of any mental disorder, (3) being the main lay healthcare supporter for the patient, (4) living in Shenzhen and having no plan to leave Shenzhen in the following 12 months after discharge and (5) being able to read text messages, answer phone calls on mobile phones, use WeChat, or any application on smart phones. All participants provided written consent before interviews, and received ¥100 (about US$15.42) to offset their efforts and time after interviews.

Patients who were with cognitive impairment that prevents providing written informed consent due to either dementia or current psychosis episodes and who were without ID, stable residence or any source of income were excluded. Particularly, patients discharged on families’ or patients’ demand against medical advice were excluded.

### Measures

#### Demographic information questionnaire

We collected socio-demographic information including gender, age, occupation, education, marriage, monthly income, and residence. For patients, we collected disease-related information including diagnosis, length of mental disorders, duration of current hospitalization. For MPs, we collected professional background information including clinical specialties, department, and years of working.

#### Interview guide questions

Experienced psychiatrists from the research team developed the interview guide questions, which were described elsewhere [48]. We adopted the following questions for this study:

- 1. What is the most difficult problem to face after discharge? (For patients and LHSs)
- 2. Reducing the risk of suicide after discharge from hospital has been a common understanding. Please explain to us why do you think there a need to focus on reducing suicide risk among psychiatric patients after discharge. (For MPs).
- 3. During the follow-up contact period after discharge, what help do you hope to get from MPs (for example, appointment scheduling, medication and follow-up reminders, assistance with applying for health insurance subsidies, introduction of preferential policies), and your LHSs? (For patients and LHSs).
- 4. Have you previously received follow-up services? What are the advantages and drawbacks of the services that you have received or are presently receiving? What are your unresolved needs? (For patients and LHSs).
- 5. How would you implement post-discharge suicide risk management from your perspective? Please briefly introduce your experience in patient suicide risk management. (For MPs).

### Data collection and quality control

We conducted interviews from August to December 2021 in private meeting rooms at SKH. Each interview was conducted by a facilitator, with a note-taker and an observer present. The note-takers were research assistants and the observers were the project leader and key members of the project team. All note-takers and observers are familiar with the hospital environment and have professional backgrounds in psychiatry. Before the interviews, the facilitator explained the purpose of the study, the purposes of the interview, and obtained written informed consent from participants, including audio recording consent. Audio recordings and field notes were transcribed into text for analysis.

We implemented strategies to increase the trustworthiness of the study. First, we contacted coordinators from different departments of SKH, who held public information sessions about the study towards inpatients before the study began. Second, interviewers were SKH faculties who were skilled with patient communication and received training about qualitative study by FH and HL. Third, considering suicide as a sensitive topic, we conducted interviews in independent, private consulting rooms and meeting rooms in SKH. This approach was adopted to minimize external disturbances, safeguard participant privacy, and ensure a relaxing interview environment. Forth, the research team listened to recorded interviews, discussed encountered challenges and difficulties, developed strategies to improve interviewers’ skills, and conduct quality control meetings to address these issues.

### Data preparation

All recordings were transcribed into text data via Iflyheard [53]. Data analysts listened to original audio recordings, read through the transcripts and field notes several times to become familiar with the content and identify initial impressions. Meanwhile, the analysts also recorded their thoughts and doubts for further analysis.

### Data analysis

We adopted thematic analysis approach to identify sub-themes and themes from interviews [54]. The coding team consisted of the experienced project leader and three data analysts as coders, the three data analysts were all graduate students and had completed training in qualitative data analysis. The data analysts had no prior associations with participants. The project leader resolved disputes, provided guidance, and performed quality control during analysis process. All researchers, encompassing interviewers, professionals responsible for formulating the guide questions, data analysts, and all authors reported the findings, were free from any conflicts of interest with the participants and the hospital.

#### Coding

We conducted a three-step coding procedure (open coding, axial coding, and selective coding) to code the qualitative data [55].

##### Open coding

The coding categories were generated through the analysis process, with the analysts initially conducting open coding. Three analysts independently identified units of analysis and performed coding, with each unit representing a portion of the transcript related to a specific topic. These units were then segmented into meaning unites, which were subsequently abstracted, and labeled as codes. The research team remained reflexivity throughout the analysis and writing process by recording, discussing, and revising established codes. Existing codes were continually modified to encompass emerging categories of meaning during coding process.

##### Axial coding

In the course of analysis, the analysts interrelated codes, deconstructed the initial structure of codes engendered through open coding, and reconfigured categories and subcategories to comprehensively elucidate the codes.

##### Selective coding

The analysts compared different categories of codes and examined their interconnections to identify a core category that could represent the main response themes to research inquiries, and which related to other categories.

### Sub-theme and theme identification

The analysts collated and grouped meaning-related codes into overarching sub-themes that represented the key ideas or patterns in the data. Then they combined related sub-themes to create the main themes. During this process, sub-themes and themes were reviewed and revised by the research team by checking for coherence, consistency, and fit with the data, as well as comparing them with the existing literature or theories.

## Results

A total number of 45 participants were recruited, including 17 patients, 8 LHSs and 20 MPs. We conducted 25 personal in-depth interviews and 3 focus group interviews. The duration of the in-depth individual interviews ranged from 16 to 64 min, with an average length of 47 min. The focus group interviews ranged from 28 to 35 min, with an average length of 32 min. Patients aged from 20 to 58 years, with a mean of 32.94 ± 10.71 years; LHSs aged from 23 to 50 years, with a mean of 39.75 ± 9.55 years; MPs aged from 25 to 51 years, with a mean of 34.15 ± 7.05 years. Details were showed in Table 2.

**Table 2:** Socio-demographic characteristics of participants

Socio-demographic information	Patients (*n* = 17)	LHSs (*n* = 8)	MPs (*n* = 20)
Gender			
Male	10 (58.8%)	2 (25.0%)	9 (45.0%)
Female	7 (41.2%)	6 (75.0%)	11 (55.0%)
Age (mean, S.D.)	32.94 (10.71)	39.57 (9.55)	34.15 (7.05)
Education			
Primary school or lower	3 (17.6%)	2 (25.0%)	0 (0.0%)
High school	5 (29.4%)	2 (25.0%)	0 (0.0%)
Bachelor	8 (47.1%)	4 (50.0%)	15 (75.0%)
Master and above	1 (5.9%)	0 (0.0%)	5 (25.0%)
Household registration			
Shenzhen	8 (47.1%)	5 (62.5%)	-
Elsewhere	9 (52.9%)	3 (37.5%)	-
Marriage			
Married	7 (41.2%)	1 (12.5%)	6 (30.0%)
Single	10 (58.8%)	7 (87.5%)	14 (70.0%)
Monthly income			
Less than 4999 Yuan	8 (47.1%)	2 (25.0%)	4 (20.0%)
5000—19,999 Yuan	6 (35.3%)	6 (75.0%)	6 (30.0%)
20,000 Yuan and above	3 (17.6%)	0 (0.0%)	10 (50.0%)
Mental disorders			
Schizophrenia	6 (35.3%)	-	-
Depression	7 (41.2%)	-	-
Bipolar disorder	4 (23.5%)	-	-
Length of mental disorders			
Less than 3 years	3 (17.6%)	-	-
3 years and above ~	14 (82.4%)	-	-
Duration of current hospitalization			
Less than30 days	10 (58.8%)	-	-
30 days and above	7 (41.2%)	-	-
Clinical specialties			
Psychiatrists	-	-	5 (25.0%)
Nurses	-	-	8 (40.0%)
Others	-	-	7 (35.0%)
Department			
Community mental health center	-	-	7 (35.0%)
Department of Emergency	-	-	7 (35.0%)
Department of Depressive Disorders	-	-	6 (30.0%)
Years of working			
Less than 5 years	-	-	10 (50.0%)
5 years and above	-	-	10 (50.0%)

“-” indicates no content

A total of 178 codes were generated, including 57 codes for post-discharge problems, and 121 codes for needs related to reducing suicide risk. Among the codes, 162 codes (91.2%) achieved consensus among all three analysts, 10 codes (5.5%) required discussion among the analysts, and 6 (3.3%) required a decision from the project leader. Details were showed in Tables 3 and 4. Only one representative verbiage for each code is featured in Tables 3 and 4, while all other participant verbiages and explanatory details are in the Supplementary additional file.

### Problems after discharge

We identified three themes related to problems patients would face after discharge: 1) problems related to self, 2) family-related problems, and 3) societal and community-related problems. Details were showed in Table 3.

**Table 3:** Identified problems after discharge

Theme	Sub-theme	Codes (n)	Examples
Problems related to self	Adaptation to daily lives	Cognition is not enough to cope with changes in life (3)	*"If one is relatively ungrown inside and still in a fragile mental state, any external change may stimulate a relapse of the disease (Patient 1)."*
		The challenge of re-adjusting to life after discharge (5)	*"… being here in the hospital is an escape from society, and I worry that when reentering society, I may experience the same feelings and state I had before being hospitalized. I am concerned that I may still have thoughts of ending my life (Patient 3)."*
		Self-isolation (3)	*"When I am taking pills, I just want to sleep during the daytime… no light in my eyes, and I smile reluctantly…can't keep my eyes open…I just don't want to contact others…I don't even feel like checking my phone messages now, and don't want to receive information from outside (patient 6)."*
	Disease-related frustration	Poor adherence to medication treatment regimen (7)	*"I stopped taking the medicine without doctors’ permission, which led to the recurrence in a short period of time (Patient 2)."*
		Failing to adjust the regimen according to the change of the disease status (3)	*"My condition got worse, because the medicine don't work anymore (Patient 13)."*
	Intimate relationship setbacks	Intimate relationship does not go well (3)	*"I have a girlfriend and we live away from each other, so it bothers me whether we can make it or not (Patient 12)."*
Family-related problems	Family conflicts	Direct family conflicts (4)	*"The cause of his relapse was that he suspected that I had touched his computer. I argued with him a few times. He finally lost his temper and hit me. My son then kicked him, he could not accept this and stopped talking to us from then on (LHS 1)."*
		Alienation from family members (6)	*"…a sense of disconnection between me and them, the family has never understood me (Patient 4)."*
		Excessive interference (4)	*"I was going to go to my girlfriend’s place, and my sister said what time I should be back, and I got really annoyed (Patient 12)."*
	Family bonding	Concern about family members (3)	*"My husband is now in the hospital and …has severe diabetes, hyperlipidemia and …kidney problems, and I am afraid of losing him (Patient 9)."*
Societal and community-related problems	Work-related problems	Stress at work (3)	*"This time I was admitted to the hospital because of anxiety at work (Patient 14)."*
		Difficulty in returning to work (4)	*" I have to communicate with my boss about what he thinks of my history of mental illness and whether it will affect my appointment (Patient 8)."*
		Difficulty in finding a new job (4)	*"I have to look for a new job after I get out (Patient 10)."*
	Problems in social connection	Difficulty in making friends (5)	*"… concern about social connection being affected. The public cognition about mental illness is not enough, so I have taken effective measures, that is, don't let other people know about my disease (Patient 1)."*
	Housing difficulty	Difficulty in finding a place to live (2)	*"… face the problem of where to live… (Patient 15)."*

#### Problems related to self

The theme encompassed three sub-themes showing how patients understood and expressed problems closely to themselves. Responses highlighted the importance of self-reliance in problem-solving as participants indicated that external efforts alone were insufficient to address these problems, highlighting the need for patients to rely primarily on themselves to find effective solutions.

##### Adaptation to daily lives

In this sub-theme, we identified several key factors contributing to adaption problems faces by patients, which highlighted the complex nature of the challenges in various aspects of patients’ daily lives after discharge. First, patients attributed their problems to a poor recognition of external world. This was evident when relapse occurred as a result of insufficient cognitive abilities to cope with changes and stress in daily live. Second, the transition from the hospital environment to life after discharge posed significant challenges for patients. This included adapting to changes in surroundings, people, events, and living habits, thus, patients consistently faced the difficulty of readjusting to family, work, and interpersonal relationships. Third, patients experienced self-isolation due to the disease, medication, and/or their own personality traits. Despite they were eager to make friends and develop intimate relationships, they did not always actively seek out to communicate with others.

##### Disease-related frustration

In this sub-theme, we identified several issues related to medication adherence among patients after discharge. Specifically, patients often struggled to take their medication regularly and on time, and unauthorized drug withdrawal was a common issue. And MPs identified this as the major cause of relapse and suicide risk*.* Side effects were a significant factor of non-adherence among patients, and, in some cases, it could lead to suicide. Patients often experienced mental status fluctuation after discharge, and it could be resulted from their failure to adjust treatment regimen as prescribed which was often due to missing further visits or appointments.

##### Intimate relationship setbacks

Patients often experienced intense loneliness and frustration when faced with intimate relationship problems, which could contribute to mental status fluctuations. These challenges were influenced by various factors, including patients' individual personalities and the impact of their illness.

#### Family-related problems

The theme encompassed two sub-themes suggesting that conflicts and relationship problems with family members would bring significant stress to discharged patients, and addressing the conflicts and promoting positive family relationships can be an important aspect for treatment and recovery.

##### Family conflicts

During the interviews, patients, LHSs, and MPs identified several factors contributing to stress and strain within the family dynamic. These factors included direct conflicts among family members, a sense of loneliness stemming from long-term lack of understanding and alienation, and excessive regulations imposed on patients. Patients frequently expressed feelings of loneliness, as they perceived a lack of understanding and support from their family members, despite knowing that they were loved. Conversely, family members often employed excessive regulations in an attempt to protect the patients, which the patients typically found annoying or burdensome.

##### Family bonding

Patients shared their concerning about family member as they perceived the distance between them and their families or worry about their health status, and the concerning may associate with their stress and mental health status.

#### Societal and community-related problems

This theme encompasses three sub-themes showing problems closely linked to the broader community and society. Addressing these requires collaborative efforts, including policy-making by relevant government departments and disease awareness campaigns led by MPs, aiming at improving patients' living conditions and overall well-being.

##### Work-related problems

Patients attributed relapses to work related factors, while they also faced challenges in returning to work for various reasons, including reduced work capacity due to illness and prolonged absence resulting from hospitalization. Additionally, patients also complained about experiencing prejudice and discrimination at workplace. It is worth noting that some patients were already unemployed prior to their admission to the hospital, while others had to give up their current job due to hospitalization. As a result, they faced the additional challenge of finding new employment after their discharge.

##### Problems in social connection

Patients expressed difficulties in making friends due to illness as well as their own personalities. Moreover, the presence of social stigma and prejudice against psychiatric patients further worsened this issue. In particular, patients often intentionally concealed their illness when making new friends, aiming to seek social interaction. However, this concealment can pose challenges in building and maintaining meaningful relationships.

##### Housing difficulty

There were patients reported having no fixed residence before being hospitalized, and they also faced difficulties in finding suitable accommodation after discharge.

### Needs for reducing post-discharge suicide

We identified four themes related to patients’ needs for reducing post-discharge suicide: 1) proactive self-management, 2) multifunctional relatives, 3) multifunctional MP group, and 4) a warm society. Details were showed in Table 4.

**Table 4:** Identified needs for reducing the risk of post-discharge suicide

Theme	Sub-theme	Codes (n)	Examples
Proactive self-management	Proactive integration	Proactively generate value (2)	"*External inevitability is beyond our power to change. I can only make internal changes, … offering help and actively listening to others. Assisting others can cultivate a sense of warmth and connection between people (Patient 1)."* *“…getting together with friends is useless (patients 13).”*
		Proactively participate in the group (5)	"* I plan to join the neighborhood dance team, who have treated me with the love and care of sisters… (Patient 9).*”
	Proactive adjustment	Proactively adjust cognition (2)	“*My cognition needs to be adjusted. What I need to overcome is myself. A change in attitude, an acceptance of things, and a correct outlook on life is the best way to reduce the risk of relapse (Patient 1)*.”
		Proactively regulate emotions (3)	" *I read a book about emotion self-help and emotion balance method… I learned to sit still and breathe deeply for 20 min to regulate my mood (patient 14)*."
	Proactive help-seeking	Proactively seek professional intervention (9)	"*I first called the emergency department and they advised me to go to the nearest hospital and I went and got a diazepam shot (patient 4)*."
		Proactively seek help from LHSs (5)	"*When I have suicidal thoughts, I ask LHS to stay with me the whole day (Patient 9).*"
Multifunctional relatives	Tangible support	Obtaining disease-related knowledge for family-based interventions (4)	"*Training or books about intervention knowledge as well as psychotherapy should be given to the patient's parents and spouse. Let them know about it and do family therapy together. Then they will be like a doctor in family, and treat the patient in case of an emergency (Patient 2)*."
		Assist patients in accessing medical resources in emergency (6)	"*As family members, we should focus on his behavior and send him to the hospital immediately if we perceive his suicidal behavior, so that further serious situations will not arise (LHS 1)*."
	Emotional support	Companionship, listening, understanding, support and encouragement (8)	"*Understanding, companionship, and support is important. If the family think that the patient is a burden to them, it will be more traumatic for the patient, who would see less hope in their life (Patient 1)*."
Multifunctional MP group	Direct professional support	Medication (15)	"*Medicine has helped me a lot on my condition, and I have learned that there are many kinds of medications for this disease, I hope to find the most suitable medication for myself soon (Patient 1)*."
		Psychotherapy (6)	"*Many people commit suicide never because they truly desire it, but rather as a means of having their concerns heard and addressed. MP group need to help patients confront the root and purpose of their suicidal ideation (Patient 8).*"
		Treatment outcome monitoring and adjustment (5)	"*Follow up regularly to observe medication intake and mental status. And there should be a feedback mechanism (MP group 2)*."
	Indirect professional support	Health education on mental illness-related knowledge (13)	"*…tell me to take medication on time, and how to deal with the disease. Give a lecture to my family about what the disease actually is and issues that the family needs to pay attention to after discharge (Patient 5)*.”
		Convenient services (15)	"*… timely medication reminders, schedule regular follow-up appointments, preferential benefits, and policy support (MP group 1)*."
	Emotional support	Companionship, listening, understanding, support and encouragement (4)	"My follow-up provider *can ask about my income or my future career plans and give me a reference (like my friend) (Patient 6)*."
A warm society	Emotional support from society and the public	Emotional support from hospital environment (5)	"*The hospital is staffed with doctors and nurses; I feel secure and protected in this small boat (Patient 9)*."
		Emotional support from work environment (4)	"*I appreciate that my boss knows about my condition and he treats me with kindness (Patient 3)*."
		Emotional support from community environment (3)	"*He needs more care, more acceptance and understanding from the community (LHS 5)*."
	Instrumental support from society	Financial help (3)	*"The government should issue a policy to provide free medication, which can help patients to take medication steadily and reduce their financial and psychological pressure (LHS 5)*."
		Employment opportunities (5)	"*A stable job gives them a security of survival, or at least a way to support themselves, which reduces their stress (LHS 5)*

#### Proactive self-management

The need of being a proactive self-encompassed three sub-themes that demonstrated participants’ willingness proactively transform themselves to enhance mental well-being and reduce the risk of suicide.

##### Proactive integration

Patients recognized that individuals may have limited control over their external environment and cannot always rely on others for assistance and support, and they pointed out that actively initiating change, helping others, expressing affection, and attentively listening to others could serve as an alternative means of gaining social support. The needs of proactive integration showed patients’ willingness to take control and to empower themselves, which they believed would facilitate their recovery. Proactive engagement in group activities and efforts to improve interpersonal relationships can foster a sense of belongingness and reduce loneliness. However, some patients expressed skepticism about the benefits of group support for their psychological well-being and showed reluctance.

##### Proactive adjustment

The sub-theme reflected patients’ belief that proactive adjusting one's cognitive processes could alleviate stress caused by discrepancies between perceived reality and actual circumstances, which would help them adapt to problems and challenges after discharge. When discussing mental health crisis, such as suicide ideation, patients mentioned taking the initiative to regulate their emotions and manage their emotional fluctuations independently.

##### Proactive help-seeking

Proactive help-seeking indicated the need for patients to share their personal emotional fluctuations with others, as such changes were often difficult to detect even by LHSs who were close to them. This necessitates patients to actively confide in others, including friends, relatives, and professional healthcare providers, to seek appropriate intervention and support. Proactively seeking professional intervention was recognized as a crucial step by both patients and MPs in situations where the risk of suicide was heightened. One patient shared his experience of seeking professional help. Patients at high-risk of suicide could benefit greatly from actively seeking help from LHSs. By proactively engaging with LHSs, patients can access. beneficial resources such as companionship and medical intervention.

#### Multifunctional relatives

The theme encompassed two sub-themes that demonstrated patients’ need for multifaceted assistance from LHSs, including emotional support and tangible support.

##### Tangible support

It was of great significance to educate LHSs on disease-related knowledge and interventions. This enabled them to understand the unique characteristics of mental illness and respect the patient's personal traits, thereby providing relatively professional care and conducting family-based interventions. Further, LHSs should closely monitor patients' condition and be aware of the resources available to help them in case of an emergency. In situations with a high risk of suicide, patients would often find it difficult to access medical intervention resources independently, making LHSs essential in providing assistance.

##### Emotional support

Emotional support could take form of companionship, active listening, understanding, encouragement, and general support, and patients highlighted the crucial role of emotional support in maintaining their hope for life.

#### Multifunctional MP group

The theme encompassed three sub themes that demonstrated participants' need for diverse aid from the MPs including professional support and emotional support.

##### Direct professional support

Direct professional services provided by MPs were widely recognized as crucial in reducing the risk of self-harm, suicide, or relapse. Participants acknowledged the importance of medication treatment and expressed their needs for MPs to prescribe appropriate and effective medication regimens. And participants considered psychotherapy to be effective for treating mental illness, particularly psychological counseling that aimed to address underlying causes of suicide ideation. Considering patients’ mental state may fluctuate rapidly in response to stressful life events, it is imperative for MPs to closely monitor patients' condition and make timely adjustments to their treatment regimen.

##### Indirect professional support

Participants believed that MPs played a crucial role in helping patients reduce the risk of suicidal self-harm and relapse; and by leveraging professional expertise and social status, they could also provide a wide range of indirect professional support besides medical treatment, especially mental health education on the information of mental illness, the importance of adherence to medication regimens, hospitalization, and follow-up care. MPs also pointed out that effective communication strategies for interacting with patients and creating safe environments were important. We noticed that patients and LHSs expected MPs to provide assistance with appointment scheduling, medication and follow-up reminders, guidance in applying for health insurance subsidies, introduction to preferential policies, transparent pricing, and the provision of a certificate of normal mental status to facilitate patients’ return to work. Nonetheless, there were patients and LHSs felt that constant reminders of their mental health condition might have a negative impact on their well-being.

##### Emotional support

In addition to offering professional support, both patients and MPs emphasized the significance of building trust and establishing a sense of intimacy during follow-up appointments. Patients expected MPs to show genuine interest in their personal traits and engage with them in a natural and friendly manner, resembling interactions with friends. By delving into the patient's inner world, demonstrating care, actively listening and accompanying the patient, and providing words of encouragement, MPs could help boost patients’ confidence and contribute to an overall improvement in their quality of life. And the display of sincere care by MPs could serves as a crucial source of motivation and upliftment for the patients, creating a positive impact on their well-being.

#### A warm society

The theme encompassed two sub themes that demonstrated the importance of a supportive and understanding society for patients’ overall welfare. This theme emphasized the importance of empathy, acceptance, tolerance, and benevolence from the society and the public towards patients, in addition to the instrumental assistance provided by the communal infrastructure, favorable governmental policies, and other factors. These elements collectively have great impact on the well-being of patients.

##### Emotional support from society and the public

A harmonious patient-patient and doctor-patient relationship within the hospital played a crucial role in providing social support for patients. Patients and LHSs reported that a significant portion of social support came from interactions with fellow patients and MPs. Emotional bonds are formed between patients, leading to mutual support. Sometimes, patients viewed the hospital as a safe haven, where they felt being protected by doctors and nurses. And they expressed preferences for the continuation of this supportive network after discharge.

Care and understanding from the work environment could bring patients a sense of kindness and warmth, contributing to their perceived social support. And participants believed that eliminating stigma and discrimination against patients in workplace and promoting correct understanding among colleagues and leaders were important in reducing patients' psychological stress after discharge.

Additionally, participants, especially MPs, demonstrated that the fear of discrimination from community neighbors often lead discharged patients to refuse follow-up care. Therefore, creating a caring and non-discriminatory community environment was crucial for patients to feel comfortable and make progress in their recovery.

##### Instrumental support from society

Patients and LHSs expressed the need for instrumental support from the society. First was financial support to help patients stay in treatment, which could alleviate the burden on patients and their families. Second was occupational support to help discharged patients reintegrate into the workplace and regain their social functioning, which aimed to facilitate their successful return to work and promote their overall well-being.

## Discussion

We deliberately employed a qualitative approach of descriptive interview as it enabled a deep exploration of participants’ personal experiences, and we identified a range of problems related to patients’ immediate surroundings at different levels: 1) problems related to self, 2) family-related problems, and 3) societal and community-related problems. We also found the unmet needs extended beyond the professional support provided by MPs and the emotional support offered by family members: 1) proactive self-management, 2) multifunctional relatives, 3) multifunctional MP group, and 4) a warm society, and post-discharge follow-up is essential to meet the unmet needs. During these follow-up contacts, MPs provide disease-related health education and empower LHSs to complete family-based intervention, as well as provide emotional and most professional support. Moreover, patients in this study acknowledged their positive roles in recovering, and we believed it shed light on help them take initiatives and implement practical steps to reduce post-discharge suicide. As far as we know, this study is one of the few studies in China that qualitatively takes a comprehensive approach involving psychiatric patients, their LHSs, and MPs. By exploring their perspectives, we addressed the post-discharge problems and unmet needs of patients in the context of post-discharge suicide management, with the goal of developing patient-centered strategies. Moreover, the same methodology of this study could be applied to examine the experiences and emotions of other populations, such as IPV victims [56].

We find that problems after discharge cross the social-ecological framework, and are similar to studies in other cultures that concerning to adaption to life, family relations, inadequate aftercare, social connection and government benefits and information [36, 57–60]. Without a doubt, the problems and challenges would increase in the severity of symptoms and the risk of post-discharge suicide, and, according to a systematic review, we believe restricting access to lethal means, medication and psychological therapy, public and physician education, and internet or hotline support are important to for discharged patients and their LHSs [61]. We believe patients, LHSs and MPs should collaborate closely to ensure adherence to medication regimens and make necessary adjustments through regular follow-ups and monitoring, and we suggest patients engage in group activities that can alleviate the overcoming feelings of self-isolation. In addition, we encourage patients to engage in psychological and therapeutic interventions that specially address personal problems they would face within intimate relationships. Meanwhile, our findings suggest family-based interventions after discharge, including education LHSs about the illness, can help foster mutual understanding between patients and LHSs and prevent family conflicts.

Our findings suggest that though emotional support provided by LHSs and professional support offered by MPs are valuable, they may not fully meet the comprehensive needs of patients and, thus, have limited effectiveness in reducing post-discharge suicide risk among patients. Firstly, from the perspectives of patients and LHSs who received mental health care services, their expectations of MPs extend beyond professional support alone. They also value emotional support from MPs, including caring, companionship, and active listening. Previous studies have reported that poor relationships between patients and clinicians were associated with increased suicide risk, thus, we believe MPs can help reduce the risk by providing both indirect professional support and emotional support that builds supportive doctor-patient relationships [19, 36]. Secondly, MPs expect patients and LHSs to better understand mental disorders so as to improve medication adherence and accept post-discharge follow-up care. However, the effectiveness of community follow-up care in reducing suicide risk has yielded inconsistent findings, likely due do different continuity of care, social and cultural factors, and potential selection bias [13, 14, 18, 27]. Nevertheless, the findings indicate that many current approaches to psychiatric aftercare are inadequate. For patients who refuse follow-up care and post-discharge management, MPs should respect their choices while strengthening pre-discharge education and providing alternative monitoring measures such as accessible intervention resources. It is also important to remind LHSs to prioritize family care. Thirdly, we notice that patients believed that LHSs equipped with the necessary knowledge and skills for family-based interventions in psychiatric care and suicide prevention can significantly contribute to reducing post-discharge suicide risk. Given the shortage of mental health resources in China, empowering LHSs with professional knowledge for family-based interventions can effectively complement some of the services that are lacking in the community [4]. Fourthly, by emphasizing the importance of patients’ self-initiatives, we believe LHSs and MPs should pay attention to increase patients’ self-empowerment that is to increase patients’ autonomy, competency over life, and relatedness by improving their extrinsic and intrinsic motivations [62, 63]. In conclusion, to effectively reduce post-discharge suicide risk among patients, it is of great importance to address the roles of LHSs and MPs in a professional yet familial manner and in a continuum of collaborative mental health services that span across communal and societal sectors, with patient-centered follow-up care at its core.

In this study, participants emphasized the tangible support from community and society, such as financial assistance, employment opportunities, fostering a warm and accepting living environment for patients. In specific, patients expressed their hope for a less stigmatizing and discriminatory community and societal environment regarding mental illnesses, which would facilitate their recovery of social functioning and improve acceptance of follow-up care. Achieving such a supportive environment largely depends on the dissemination and promotion of knowledge about mental illnesses by MPs and the media at a broader societal level. These findings were aligned with evidence-based guidelines, which provide a strong foundation for promoting effective mental health care and suicide prevention [40–42]. However, we acknowledge the implementation gap between the guidelines and the current reality, and we encourage efforts from sectors of health, social welfare, social safety, public affairs, and governance etc., to bridge the divide.

### Limitations

We must consider the limitations of this study. Firstly, due to the COVID-19 pandemic, we were unable to conduct focus group interviews and LHSs faced limitations in accessing the hospital's in-patient area, which led to a smaller sample size of LHSs than initially anticipated (8 vs 15). Thus, we acknowledge that the conclusions drawn from this study may not fully capture the viewpoints and perspectives of LHSs. Secondly, we acknowledge that this study recruited patients with specific diagnoses, such as depression, bipolar disorder, and schizophrenia, who are known to be at a higher risk of post-discharge suicide; and we might have missed perspectives on their needs in community mental health care services from patients with other mental disorders. Thirdly, we noticed some participants (patients and LHSs) did not fully express their perspectives even after prompt questions, and we assume that they might have never talked about problems and needs related to post-discharge suicide before. Thus, we encourage future studies pay specific attention to this group of patients and their LHSs. Forth, as a qualitative study, we did not conduct in-depth analyses to explore potential variations in problems and needs for reducing post-discharge suicide among participants with different socio-demographic background due to a small sample size. We encourage future studies to fill this gap, especially focusing on patients with non-severe mental disorders. Fifth, we excluded patients who were discharged against medical advice, with cognitive impairment, with no ID, stable residence nor any source of income regard to the feasibility of executing the bigger implementation study. Nevertheless, we recognize that the needs for mitigating suicide risk in these subgroups may diverge from those of participants included in our study. Their input also bears significance in enhancing both clinical and community services and warrants further study.

Our findings have important implication for developing strategies against post-discharge suicide embedded in a self-family-MPs-community and society support system. It is important to help patients hold correct understandings of their diseases and interventions that enable them to maintain a proactive attitude. Within families, LHSs should express their respect and understandings towards patients, provide essential emotional support and acquire knowledge about diseases to provide effective family-based interventions.

## Conclusions

In this paper, we share a unique perspective on the problems and needs related to post-discharge suicide as perceived by psychiatric patients, LHSs and MPs. The findings highlight several key areas where patients face significant difficulties, including cognitive problems, outlook on life and the world, family conflicts and bonding, social communication, employment, and housing issues. Their unmet needs concerning to stakeholders including themselves, their LHSs, MPs as well as the broader community and society. The results indicate future interventions should involve all stakeholders as a cohesive system. Patients should be actively involved in the development of interventions, taking an empowered role in their own care. LHSs should acquire enhanced professional knowledge to effectively facilitate family-based interventions. MPs, in addition to providing professional support, should provide emotional support and some convenient services for patients and their families as well. Furthermore, media, policymakers, and the public should collaborate in creating a supportive environment that promotes patients' well-being and reduce the post-discharge suicide risk.

### Supplementary Information

**Additional file 1.**

## Data Availability

The interview datasets generated and analyzed during the current study are not publicly available due to their sensitive, identifiable nature and the assurances given to study participants but all analysis codes and research materials are available from the corresponding author on reasonable request.

## Abbreviations

LHS: Lay healthcare supporter
MP: Mental health professional
CBPR: Community-based participatory research
