# Supplementary material for: Preventing post-discharge suicides in psychiatric patients: insights from patients, lay healthcare supporters, and mental health professionals—a qualitative analysis
Source: BMC Public Health. 2024 Jan 2;24:64. doi: 10.1186/s12889-023-17475-w (PMC10762919; doi:10.1186/s12889-023-17475-w)
Supplement: Supplementary file 1 — Additional file 1. [file 12889_2023_17475_MOESM1_ESM.docx]

**Appendix 1. Problems after discharge**

***Problems related to self***

**1. Adaptation to daily lives**

***(1)*** ***Cognition is not enough to cope with changes in life.*** Patients identified their poor cognation of external world as root of their problems. The relapse occurs when their cognation is not enough to cope with changes in everyday life, which cause great stress. For example, one participant said: "*If one is relatively ungrown inside and still in a fragile mental state, any external change may stimulate a relapse of the disease (Patient 1)*." While, some participants identified the problem as the perspective of living, "*My cognition, that is, the way of looking at a thing is still problematic, and this is the most fundamental problem (Patient 2)*." And, the changes could come from relationship, work, and study, "…*fell in love, then broke up, and academic setbacks (Patient 4).*"

***(2) The challenge of re-adjusting to life after discharge.*** Due to the differences between life inside the hospital and after discharge, concerning surroundings, people, events and living habits, patients always face the challenge of re-adjusting to family, work and interpersonal relationships after discharge. As one patient demonstrated that "*My being here (in hospital) now is like escaping from society, and reentering society may come back to the feeling and state I was before hospitalization, and I may still want to end my life (Patient 3)*." This challenge was also identified by LHSs: "*He has been in the hospital for several months and may have dependency, and he can't take care of himself after he is discharged from the hospital (LHS 6)*." One LHS emphasized the great challenge of facing with unprepared life events: "*As long as there are no accidents or he is not told something unexpected, he is normal. If there is a little bit of a big emergencies, he may not adapt very well (LHS 7)*."

***(3) Self-isolation.*** Patients are in self-isolation due to the disease, medication and their own personality, eager to make friends and build intimate relationships while often do not actively seek to communicate with others. As one patient said: "*When I am taking pills, I always just want to sleep during the daytime. There is no light in my eyes, and I smile reluctantly, I can't keep my eyes open when I take pictures with friends, and I just don't want to contact many people…I don't even feel like checking my phone messages now, I don't want to receive information from the world (patient 6).*" LHSs perceived the same way: "*Since he got this disease, we have felt that he was gradually detached from society (LHS 2).*" And "He prefers to stay at home and doesn't spend much time outside *(LHS 5)*."

**2. Disease-related frustration**

***(1) Poor adherence to medication treatment regimen.*** Patients, LHSs all mentioned that patients could not take medicine regularly and on time after discharge and unauthorized drug withdrawal: "*I stopped taking the medicine without authorization, which led to the recurrence in a short period of time (Patient 2)*." And "*He wasn't very adherence to medication treatment regimen. We were worried that he might stop taking the medication without us knowing about (LHS 4)*." And MPs identified this problem as the major cause of relapse and suicide-related behaviors: "*Failing to adhere to medication regimen is an important reason for the increased risk of suicide, relapse and re-admission (MP group 2).*" And "*Many patients having poor adherence to medication regimen are at risk of unstable mental health conditions (MP group 3)*."Side effect is significant reason for patients to stop taking medicine or taking irregularly. "*After taking pills for half a year, I felt that this medicine would hurt my body and my stool was a little dry after eating it, so I gradually reduced it and stopped taking it when I found nothing happened (Patient 9)*." This is verified by MPs: "*Adverse side effects after discharge may lead to poor medication compliance, aggravation of illness and suicide (MP group 3)*."

***(2) Failing to adjust the regimen according to the change of the disease status.*** Many patients facing a mental status fluctuation after discharge. However, they fail to adjust treatment regimen because of absence from further visit or other reasons. As one patient said: "*My condition got worse and the original medicine didn't work (Patient 13).*" And a LHS identified that "*He took medicine for a long time, but the effect was not obvious (LHS 2)*."

**3. Intimate relationship setbacks.** Due to multiple reasons such as their own personality and illness, patients may experience intense loneliness and frustration when dealing with intimate relationship problems, which is likely to cause mental status fluctuation. For example, one patient said: "*I have a girlfriend and we are in different places, so it bothers me whether we can make it or not (Patient 12)*." Another patient faced intimate relationship setbacks said: "*I’m gonna find my ex-girlfriend back after discharge (Patient 15)*." This problem was perceived by LHS as well: "*He is quite sensitive, and the first few relapses were because he was not very successful in his relationship or had an argument with his girlfriend, and it was easy to relapse (LHS 5)*."

***Family-related problems***

**1. Family conflicts.** Patients, LHSs and MP group all identified that direct family conflict, loneliness due to long-term incomprehension and alienation of family members, and excessive regulation cause a lot of psychological stress to patients.

***(1) Direct family conflicts.*** Direct conflicts with family members have a great impact on patients’ mental status. As one patient said: "*My husband smashed things at home during my postnatal recovery period and scolded me for a week, and my mother-in-law was present and did not say a word. I was really angry with this mother and son, and was hospitalized many times over and over again because of them (Patient 17)*." Another LHS describe one direct conflict between the patient and family members: "*The cause of his relapse this time was that he suspected that I had touched his computer. I argued with him a few times. He finally lost his temper and hit me. My son then kicked him, he could not accept it and stopped talking to us from then on (LHS 1)*." MPs also emphasized potential family conflict as problem faced with patients after discharge: "*The patient mishandled interpersonal relationships at home and returned to the original stimulating environment after discharge (MP group 3)*."

***(2) Alienation from family members.*** A lot of patients often feel alienated from their family members, that they are ununderstood and unsupported, which increases their feelings of loneliness and disconnection. Just as one girl said: "My mother and I have a big age difference, so there is a generation gap. She does not understand the tattoo industry that I work in (Patient 5)." And even though they can feel love, they are still surrounded by loneliness, lacking and desiring for understand. "Although I can see that they really love me, but there is always a sense of disconnection between me and them, the family has never understood me (Patient 4)."

1. ***Excessive interference.*** Excessive regulation from family members prevents patients from completing the intended action and is identified as an annoying factor: "*I was going to go to my girlfriend’s place, and my sister said what time I should be back, and I got really annoyed (Patient 12)*." Excessive interference may cause potential family conflict: "*I’ve grown up and have my own views. I can't follow my parents' ideas all my life (Patient 6)*."

**2. Family bonding.** The distance between patients and their families and the worry and concern about their health status aggravates patients’ mental stress. Some may face the possibility of losing the closest person: "*My husband is now in the hospital and I am worried about him because he has high diabetes, hyperlipidemia and hypertension, and it was found that he has kidney problems and I am afraid of losing him (Patient 9)*." Some may concern about the family: "*I am too far away from my son, he is in Fujian Province and I am in Guangdong Province (Patient 16)*." or worry about their health: "*I'm worried about my mom’s relapsing again (Patient 8)*."

***Societal and community-related problems***

**1. Work-related problems**

***(1)*** ***Stress at work.*** Many patients face pressure at work process, which is a cause of previous relapses: "*This time I was admitted to the hospital because of anxiety at work (Patient 14)*."

***(2) Difficulty in returning to work.*** Many patients faced difficulties in returning to work for various reasons, such as reduced competence to work due to illness and prolonged absence from work due to hospitalization: "*I don't know if I will be able to do my previous job under my current condition (Patient 12)*." or prejudice against people with mental illness in the workplace. " *I have to have a conversation with my boss about what he thinks of my history of mental illness and whether it will affect my future appointment (Patient 8)*."

***(3) Difficulty in finding a new job.*** Many patients were unemployed before they were admitted to the hospital, or they give up their current job because of hospitalization and face the difficulty of finding a new job after discharge: "*I'm going to look for a new job after I get out (Patient 10)*."

**2. Problems in social connection.** Many patients have difficulty making friends due to illness and their own personalities, as well as the social stigma and prejudice attached to mental illness, with particular reference to the tendency of patients to deliberately hide their illness when making friends in order to seek interaction with others. For example, one patient said: "*There would be a concern about that social connection being affected after discharge. Because the public cognition about mental illness is at a low level, so I have taken effective measures, that is, don't let other people know about my disease (Patient 1)*." Another patient found it hard to establish social connection with people around him: "*I have no friend by my side, just online chats. I'm always alone (Patient 4)*."

**3. Housing difficulties.** One patient confided that he had no fixed residence before hospitalization and faced difficulties in finding accommodation after discharge: "*… face the problem of where to live after being discharged from the hospital (Patient 15)*

**Appendix 2. Needs for reducing suicide risk**

***Proactive self-management***

**1.Proactive integration**

***(1) Proactively generate value.*** Recognizing that individuals have limited control over the external environment and often cannot rely on others for assistance and support, patients understand that actively initiating change, offering aid to others, expressing affection, and attentively listening to others is an alternative means of gaining social support. This approach is proactive, controllable, and more effective for one's own healing. As one patient articulated, "*External inevitability is beyond our power to change. I can only make internal changes, such as offering help and actively listening to others in the workplace. Assisting others can cultivate a sense of warmth and connection between people (Patient 1)."*

***(2) Proactively participate in the group.*** Proactive engagement in group activities and efforts to improve interpersonal relationships can foster a sense of belonging and reduce feelings of loneliness. As one patient shared, "*Upon my discharge from the hospital, I plan to join the neighborhood dance team, who have treated me with the love and care of sisters... Although I previously viewed playing mahjong as a waste of time, during my hospitalization, I learned to play and now recognize its value as a popular pastime for older adults (Patient 9).*” Another patient emphasized the importance of socializing with colleagues, stated, “*Gathering for birthday dinners has helped us develop closer relationships (Patient 16).*” Spending time with family members is also identified as a source of comfort: “*I have learned to communicate more with my family members and live with them (Patient 10).*” Some patients mentioned keeping contact with one another and planned to continue socializing after discharge: “*We (patients) have exchanged contact information and plan to have dinner together (Patient 6).*” The beneficial effects of participating in group activities were acknowledged by LHSs as well. For example, one healthcare provider stated, “*Interacting with a larger number of colleagues can foster closer connections with others... Additionally, participating in outdoor activities such as mountain climbing on Sundays can also have a positive impact (LHS 5).*” However, some patients expressed skepticism about the benefits of group support for their psychological well-being and were less inclined to participate actively: “*getting together with friends is useless (patients 13)*”

**2. Proactive adjustment**

***(1) Proactively adjust cognition.*** Proactive adjustment of one's cognitive processes can alleviate stress caused by discrepancies between perceived reality and actual circumstances. Patients have come to realize the importance of aligning their cognition with reality and adopting an appropriate perspective to view external changes when faced with challenges. As one patient said: “*A lot of my ideas and mentality need to be adjusted. What I need to overcome is myself. A change in attitude, a better state of mind, an acceptance of things outside, and a correct outlook on life and values is the best way to reduce the risk of relapse (Patient 1)*.” Another patient emphasized the significance of modifying cognition towards society and events, stating, “*Changing one's perception of society and things is crucial and addresses the root causes of psychological distress (Patient 2).*”

***(2) Proactively regulate emotions.*** In high-risk situations such as suicidal ideation, patients take the initiative to regulate their emotions and control their emotional fluctuations independently. As one patient stated: " *I read a book about emotion self-help and emotion balance method, which is effective. I learned to sit still and breathe deeply for 20 minutes to regulate my mood (patient 14)*." Another patient mentioned: "*When I was in a bad mental state for several days and had auditory hallucinations and suicidal ideation, I would try to divert my attention through watching TV and listening to music (patient 5)*."

**3. Proactive help-seeking.** Proactive help-seeking involves patients taking initiative to share their personal inner mood fluctuations with others, as such changes are often difficult to detect even by LHSs who are in close proximity to them. This necessitates patients to actively confide in others, including friends, relatives, and professional healthcare providers, in order to seek appropriate intervention and support.

***(1) Proactively seek professional intervention.*** Proactively seeking professional intervention is recognized as a crucial step by both patients and MPs in situations where the risk of suicide is heightened. One patient had shared his experience of seeking professional help: "*I first called the emergency department and they advised me to go to the nearest hospital and I went and got a diazepam shot (patient 4)*." Patients’ access to professional intervention also relies on health education and dissemination of relevant knowledge by MPs: "*There is a crisis intervention hotline, through which patients can seek professional help when they are unable to cope on their own (MP group 2)*."

***(2) Proactively seek help from LHSs.*** Patients who find themselves in high-risk suicidal situations can benefit greatly from actively seeking help from LHSs. By proactively contacting family and friends, patients can access helpful resources such as companionship or medical intervention with their help. For example, one patient expressed the need for 24-hour company from LHS, stating: "*When I have suicidal thoughts, I ask them to stay with me and not leave for the whole day (Patient 9).*" LHSs have also recognized the importance of patients reaching out to them for help, with one LHS stating that "*He would reach out to me and say that he was feeling in a bad state lately and was having hallucinations (LHS 5).*"

***Multifunctional relatives***

**1. Tangible support**

***(1) Obtaining disease-related knowledge for family-based interventions.*** Educating LHSs on disease-related knowledge and intervention methods is of great significance. As one patient stated: "*Training or books about intervention knowledge as well as psychotherapy should be given to the patient's parents and spouse. Let them know about it and do family therapy together. Then they will be like a family doctor, and treat the patient in case of an emergency (Patient 2)*." This would enable LHSs to comprehend the unique characteristics of mental illness and respect the patient's personal traits, thereby enabling them to provide relatively professional care and conduct family-based interventions. The perspective from LHSs confirms the need: "*Education is definitely necessary, we need to have a preliminary knowledge of disease and understanding of the patient and give him a proper guidance* *(LHS 7)*." Family-based interventions conducted by LHSs could also serve as an extension of inpatient treatment and facilitate patients' transition from receiving inpatient treatment to community living: "*Some LHSs do not have a good understanding of depression and do not pay enough attention to it. After LHSs obtain enough knowledge about disease, the management within hospital can be carried over to family, which is a good way to improve suicide prevention in the community (MP group 1)*." Moreover, this education would facilitate MPs to conduct further follow-up care: “*The lack of understanding about the importance of follow-up services results in the rejection of follow-up personnel, thereby greatly compromising the quality of follow-up services we provide …Tell the family the importance of follow-up (MP group 3)*.”

***(2) Assist patients in accessing medical resources in emergency.*** LHSs should closely monitor the patient's condition and be aware of the resources available to help them in case of an emergency. In situations of high risk of suicide, it is often difficult for patients to access medical intervention resources on their own, thus making LHSs essential in offering assistance. One patient stated that "*In cases of self-harm and suicide, LHSs should immediately accompany the patient to the hospital (Patient 13)*." And "*As LHSs, we should observe his behavior and send him to the hospital immediately if we perceive his suicidal behavior, so that further serious situations do not arise (LHS 1)*." MPs also suggested that" *If the family identifies warning signs of suicide after the patient's discharge from the hospital, they should contact the us and take the patient to the emergency room for immediate medical attention (MP group 3)*."

**2. Emotional support.** Emotional support, a traditional family function, is a useful form of support that families can offer. Emotional support can be in the form of companionship, active listening, understanding, encouragement, and general support. One patient articulated the crucial role of emotional support in maintaining their hope for life: "*Understanding, companionship, and support are important. If the family think that the patient is a burden to them, it will be more traumatic for the patient, who would see less hope in living. Because even the closest people do not understand the suffering inside of me (Patient 1)*." As one LHS said:"…*be there for him, take care of him (LHS 7)*."And MPs highlighted emotional support from family: "*The most pressing need for mitigating the risk of suicide is still the family's support, understanding the patient's experience, recognizing their suffering, and not giving up on them, so that they have the support to continue living (MP group 1)*."

***Multifunctional MP group***

**1. Direct professional support.** The direct professional services offered by MP group are widely recognized as one of the most essential needs to reduce the risk of self-harm, suicide or relapse.

***(1) Medication.*** Participants affirmed the important role of medication treatment and expressed their needs for MPs to provide appropriate and effective medication regimens: "*Medicine has helped me a lot on my condition, and I have learned that there are many kinds of medications for this disease, I hope to find the most suitable medication for myself in a short time (Patient 1)*." As well as emergency medication regimens used under situations where the risk of suicide is increased: "*The doctor prescribes an emergency medication regimen, which my family and I are cognizant of, and it can be efficacious in regulating my emotions during an emergency (Patient 1)*." An LHS also said: " *As nonprofessionals, we LHSs can merely send an individual in a poor mood to the hospital, where a physician can intervene with medication to regulate their mood (LHS 5)*." The MP group believed the importance of medication adherence in mitigating the risk of self-harm and suicide: "*Mental illness is a chronic condition, and the reduction and discontinuation of medication may cause severe mood swings (MP group 2)*."

***(2) Psychotherapy.*** Psychotherapy is considered an effective form of therapy for mental illness, particularly when it includes psychological counseling aimed at addressing the underlying causes of the illness, such as in the case of suicidal ideation: "*Many people commit suicide never because they truly desire it, but rather as a means of having their concerns heard and addressed. MP group need to help patients confront the root causes and purpose of their suicidal thoughts (Patient 8).*" One LHS expressed the need for emotion relaxing after discharge: "*It's best to have a professional counselor come over every half month to ease his emotions (LHS 8)*." MPs also confirmed that: "*Psychotherapy for patients is an urgent need (MP group 3)*."

***(3) Treatment outcome monitoring and adjustment.*** The mental condition of patients with mental illness may fluctuate rapidly in response to stressful events in their lives. Therefore, it is imperative for MPs to closely attend to the patient's condition, and make timely adjustments to the treatment regimen. Just as one patient stated: "*Patients require tailored treatments and medication doses in response to their unique conditions, and MPs must engage in effective monitoring, communication, and make necessary treatment adjustments based on feedback received about the treatment outcomes (Patient 1)*." MPs may conduct monitor and adjustment with the help of LHSs: "*MPs can send me a questionnaire to collect information on the patient's recent condition in order to better provide medical services adjustments (LHS 8)*." MPs also recommended implementing a feedback mechanism: "*Follow up regularly to observe medication intake and mental status. And there should be a feedback mechanism (MP group 2)*."

**2. Indirect professional support.** MP groups play a crucial role in helping patients mitigate the risk of suicidal self-harm and relapse. Leveraging their professional expertise and social status, they can offer a range of indirect professional supports in addition to providing direct treatment to patients.

***(1) Health education on mental illness-related knowledge.*** Education is essential for improving adherence to medication and treatment regimens. This includes educating patients and LHSs on the characteristics of mental illness, the importance of adhering to medication regimens, hospitalization, and follow-up care: "*…Tell me to take medication on time, some ways to deal with the disease. Give a lecture to my family about what the disease actually is and issues that the family needs to pay attention to after discharge (Patient 5)*.” Effective communication strategies for interacting with patients and creating safe environments are also important: "*Advise them how to communicate with patients, to recognize some warning signs for suicide and how to deal with them when they appear (MP group 3)*." Additionally, recognizing warning signs of suicide and knowing how to intervene during emergency situations is critical: "*We need news related to the disease or explanation of the disease, popularizing the knowledge of the disease and some guidance on how to face it (LHS 7)*." To increase patient adherence to treatment, health education programs for LHSs can be effective: "*The family may not approve of our treatment, and they need authoritative education and guidance to improve their knowledge of the disease, and they may be more cooperative with our work (MP group 2)*." Furthermore, promoting a correct understanding of mental illness among the public could improve overall mental health outcomes: “*There is still a lack of understanding of the disease in society, and it seems that they have made depression a popular experience, as if anyone who has the disease is in great need of pity from others. That made me not want to take medication and not want people to know that I was ill. MP group should make the public understand what the disease is through official accounts, which is a great help to us patients (Patient 1)*."

***(2) Convenient services.*** The convenience services provided by MP Group, including appointment scheduling, medication and follow-up reminders, assistance with applying for health insurance subsidies, introduction of preferential policies, transparent pricing, and the provision of a certificate of normal mental status to aid patients in returning to work, are beneficial in improving their mental well-being. For example, one patient stated his need for medication delivery: "*It is usually troublesome to get the medicine, I hope the medicine will be directly delivered to our home (Patient 14)*." Another patient described her need for convenient emergency registration service: "*At that time (with warning signs for suicide), I was keen to register myself for treatment in order to safeguard my life. Unfortunately, no such avenue is available, and therefore, I am hopeful that the hospital will provide accessible registration services (Patient 1)*." MP group has been made aware of this: "*Provide him with timely medication reminders, schedule regular follow-up appointments, offer preferential benefits, and provide policy support, among other measures (MP group 1)*." Nonetheless, a handful of patients and LHSs expressed concern that the medication and follow-up reminders after discharge might trigger psychological distress, as it could constantly remind them of their mental health condition: "*He (the patient) actually exhibits resistance towards engaging with these medical professionals and is averse to meeting with them. It would be stressful to be reminded of a follow-up visit (LHS 4)*."

**3. Emotional support.** In addition to offering professional support, both patients and MP group emphasized the significance of building trust and establishing a sense of intimacy during follow-up appointments: "*I may be concerned about what I look like to you (the MP) and want you to tell me about it (Patient 8).*" Patients desire that the MPs take an interest in their personal traits and converse with them in a natural and friendly manner, “just like friends”: "*MP group can ask about my income or my future career plans and give me a reference (Patient 6)*." By delving into the patient's inner world, demonstrating care, actively listening and accompanying the patient, and providing words of encouragement, the medical professionals help boost the patient's confidence and contribute to an overall improvement in their quality of life: "*Once a trusting relationship is established with patients, they will trust the doctor, open up to the doctor, and be cooperative with the treatment (MP group 1)*." The display of sincere care by MPs serves as a crucial source of motivation and upliftment for the patients: "*We really need care from the doctor via phone call and need that kind of emotional support (Patient 3)*."

***A warm society***

**1. Emotional support from society and the public**

***(1) Emotional support from hospital environment.*** Harmonious and friendly patient-patient and doctor-patient relationship within the hospital are a pivotal constituent of social support for patients. Many patients and LHSs reported that a large part of patients' social support comes from other patients and MP group. Patients have formed emotional bonds with each other, and the subsequent mutual support has taken place: "*I have made new friends since being hospitalized and they have helped me (Patient 6)*." Some patients view the hospital as a safe haven where they receive protection from doctors and nurses: "*While the hospital is staffed with doctors and nurses, I feel secure and protected in this small boat (Patient 9)*." And they prefer the continuation of this support after discharge: "*He still needs to be treated and cared for by MP group after hospitalization (LHS 5)*."

***(2) Emotional support from work environment.*** Care and understanding from the work environment brings patients the experience of kindness and warmth so that they access more self-perceived social support: "*I appreciate that* *the boss of the place where I work knows about my condition and he treats me very well and with kindness (Patient 3)*." The elimination of mental illness stigma and discrimination in the workplace and the correct understanding of mental illness by colleagues and leaders are important in reducing patients' psychological stress. One patient articulated their worry about workplace discrimination and need of elimination of mental illness stigma and discrimination by the dissemination of knowledge about mental illness. "*I need to communicate with my leader about how he views my psychiatric history, whether it will affect my appointment in the future, and eliminate the misconceptions about mental illness, and I wonder if you (the hospital) can make a video or a documentary and give a lecture to dispel people's fear of mental disorders (Patient 8)*."

***(3) Emotional support from community environment.*** Many people refuse follow-up care because they are concerned about the discrimination from their community neighbors: "*I was worried about privacy disclosure, so I gave up the medical insurance subsidy and follow-up visits (LHS 3)*.” And MPs confirmed that "*The patients doesn't want the fact that they are hospitalized here to be exposed, and they don't want to be known by their community, because they don't want to receive discrimination from some neighbors and friends (MP group 2)*." Therefore, providing a caring and non-discriminatory community environment is crucial for patients to feel comfortable and make progress in their recovery. And patients and LHSs really appreciate it: "*There is kindness in the community and people who know about my condition give me a lot of support (Patient 3)*." "*He needs more care, more acceptance and understanding from the community (LHS 5)*."

**2.Instrumental support from society**

***(1) Financial help.*** Patients and LHSs expressed the need for financial help from the community to sustain treatment: "*I need financial support (Patient 11).*" and "*Of course I hope there will be subsidies for this disease (Patient 12)*." Economic assistance can alleviate the burden on patients and effectively reduce the risk of suicide: "*The government should have a policy to give them free medication, which can help patients to take medication steadily for a long time and reduce their financial and psychological pressure (LHS 5)*."

***(2) Employment opportunitie***s. Employment plays a crucial role in restoring the social functioning of patients. Just as one patient said: "*The support provided through employment is of utmost importance to me, as I cannot imagine my life without a job (Patient 6)*." By obtaining economic resources and being distracted from negative moods, patients are able to develop self-worth and obtain new friendship support in work environment: "*A stable job or employment opportunity that gives them a security of survival, or at least a way to support themselves, which reduces their stress (LHS 5)*." MPs have also affirmed the crucial role of work: "*Many patients cannot restore their social functions after discharge, mainly because they want to come to work, but are discriminated against, and they cannot find a job as a financial support for themselves, so the risk of relapse and suicide will always exist (MP group 1)*.
